# *trans*-Diaqua­bis(ethyl­enediamine-κ^2^ *N*,*N*′)copper(II) bis[3-(3-pyrid­yl)propionate] dihydrate

**DOI:** 10.1107/S1600536808005400

**Published:** 2008-03-05

**Authors:** Jan Moncol, Peter Segľa, Dušan Mikloš, Andreas Fischer, Koman Marian

**Affiliations:** a Department of Inorganic Chemistry, Slovak Technical University, Radlinského 9, SK-812 37 Bratislava, Slovakia; b Inorganic Chemistry, Royal Institute of Technology, 100 44 Stockholm, Sweden

## Abstract

The asymmetric unit of the title complex, [Cu(C_2_H_8_N_2_)_2_(H_2_O)_2_](C_8_H_8_NO_2_)_2_·2H_2_O, contains one anion, one half-cation and one water mol­ecule. The Cu^II^ atom in the [Cu(en)_2_(H_2_O)_2_]^2+^ cation (en is ethyl­enediamine) lies on an inversion centre. The four N atoms of the en ligands in the equatorial plane around the Cu^II^ atom form a slightly distorted square-planar arrangement, while the slightly distorted Jahn–Teller octa­hedral coordination is completed by two water O atoms in axial positions. In the crystal structure, intra- and inter­molecular N—H⋯O and O—H⋯O hydrogen bonds form a three-dimensional network.

## Related literature

For general background, see: Hathaway & Hodgson (1973 ▶); Bernstein *et al.* (1995 ▶); Janiak (2000 ▶); Jeffrey (1997 ▶). For similar structures, see: Jašková *et al.* (2007 ▶); Mimino­shvili *et al.* (2005 ▶); Carballo *et al.* (2005 ▶); Segla *et al.* (2000 ▶); Liu *et al.* (2004 ▶); Sharma *et al.* (2005 ▶); Anacona *et al.* (2002 ▶); Emsley *et al.* (1988 ▶, 1990 ▶); Li *et al.* (2005 ▶); Gonzalez-Alvarez *et al.* (2003 ▶); Lee *et al.* (2005 ▶); Mahadevan *et al.* (1986 ▶); Kovbasyuk *et al.* (1997 ▶); Harrison *et al.* (2007 ▶).
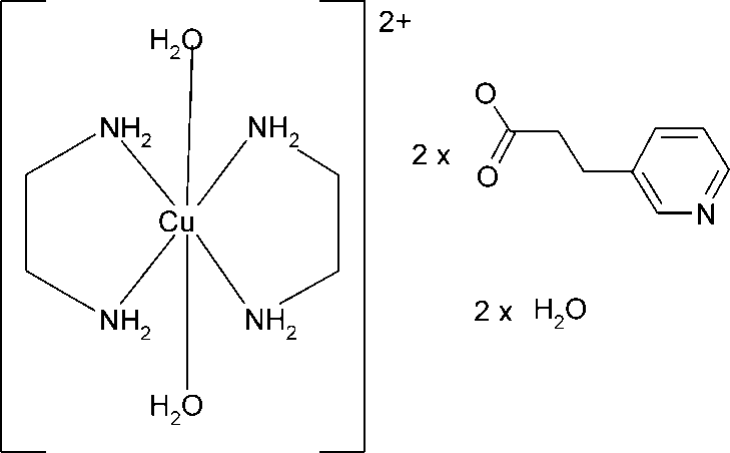


## Experimental

### 

#### Crystal data

[Cu(C_2_H_8_N_2_)_2_(H_2_O)_2_](C_8_H_8_NO_2_)_2_·2H_2_O
*M* *_r_* = 556.13
Triclinic, 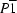

*a* = 6.2620 (1) Å
*b* = 8.5660 (2) Å
*c* = 13.3550 (4) Å
α = 75.271 (1)°
β = 83.809 (1)°
γ = 70.863 (1)°
*V* = 654.30 (3) Å^3^
*Z* = 1
Ag *K*α radiation
μ = 0.47 mm^−1^
*T* = 153 (2) K
0.45 × 0.25 × 0.20 mm

#### Data collection

Bruker–Nonius KappaCCD diffractometer
Absorption correction: numerical (*HABITUS*; Herrendorf & Bärnighausen, 1997 ▶) *T*_min_ = 0.808, *T*_max_ = 0.915
15039 measured reflections
2989 independent reflections
2644 reflections with *I* > 2σ(*I*)
*R*_int_ = 0.079

#### Refinement

*R*[*F* ^2^ > 2σ(*F* ^2^)] = 0.041
*wR*(*F* ^2^) = 0.086
*S* = 1.07
2989 reflections
160 parameters
H-atom parameters constrained
Δρ_max_ = 0.38 e Å^−3^
Δρ_min_ = −0.31 e Å^−3^

### 

Data collection: *KappaCCD Software* (Nonius, 1997 ▶); cell refinement: *SCALEPACK* (Otwinowski & Minor, 1997 ▶); data reduction: *SCALEPACK* and *DENZO* (Otwinowski & Minor, 1997 ▶); program(s) used to solve structure: *SIR97* (Altomare *et al.*, 1999 ▶); program(s) used to refine structure: *SHELXL97* (Sheldrick, 2008 ▶); molecular graphics: *ORTEP-3* (Farrugia, 1997 ▶); software used to prepare material for publication: *enCIFer* (Allen *et al.*, 2004 ▶).

## Supplementary Material

Crystal structure: contains datablocks I, global. DOI: 10.1107/S1600536808005400/hk2427sup1.cif

Structure factors: contains datablocks I. DOI: 10.1107/S1600536808005400/hk2427Isup2.hkl

Additional supplementary materials:  crystallographic information; 3D view; checkCIF report

## Figures and Tables

**Table 1:** Hydrogen-bond geometry (Å, °)

*D*—H⋯*A*	*D*—H	H⋯*A*	*D*⋯*A*	*D*—H⋯*A*
N1—H1*A*⋯O2^i^	0.92	2.32	3.130 (3)	147
N1—H1*A*⋯O1^i^	0.92	2.41	3.247 (2)	152
N1—H1*B*⋯O2*W*	0.92	2.11	2.944 (3)	151
N2—H2*A*⋯O1^ii^	0.92	2.29	3.150 (3)	155
N2—H2*B*⋯O1	0.92	2.12	3.019 (2)	164
O1*W*—H1*W*⋯O2^i^	0.84	2.06	2.873 (3)	164
O1*W*—H2*W*⋯O1	0.84	2.00	2.814 (2)	164
O2*W*—H3*W*⋯N3^iii^	0.84	2.08	2.899 (3)	163
O2*W*—H4*W*⋯O2^iv^	0.84	2.03	2.859 (3)	169

Symmetry codes: (i) 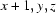; (ii) 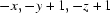; (iii) 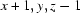; (iv) 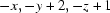.

**Fig. 1.**
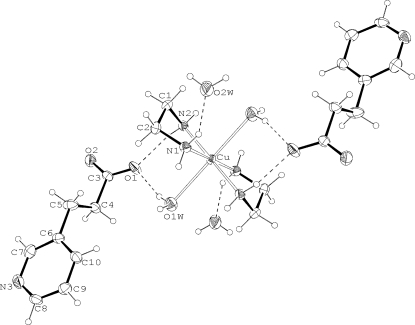
The molecular structure of the title molecule, with the atom-numbering scheme. Displacement ellipsoids are drawn at the 30% probability level.

**Fig. 2.**
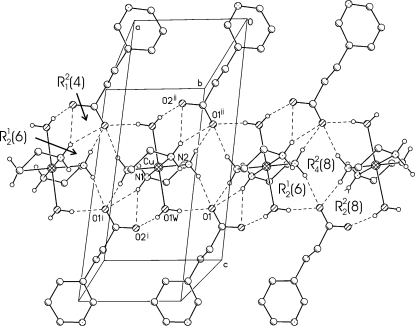
Part of the crystal structure of (I), showing the formation of R21(4), R12(6), R24(8) and R22(8) motifs [symmetry codes: (i) x + 1, y, z; (ii) -x, -y + 1, -z + 1].

**Fig. 3.**
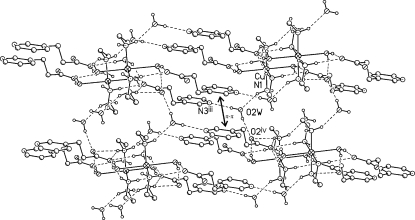
A packing diagram of (I), showing hydrogen bonds and π-π stacking interactions, as dashed lines [symmetry codes: (iii) x + 1, y, z - 1; (iv) -x, -y + 2, -z + 1].

## supplementary crystallographic information

### Comment

It is well known that copper(II) complexes having ethylenediamine (en) ligands show flexible coordination environment and adopt semi-coordation with tetragonal distortion. As part of our efforts to investigate metal(II) complexes based on pyridyl-carboxylic acids, we report herein the crystal structure of the title compound, (I).

The asymmetric unit of (I), (Fig. 1), contains one anion, one half-cation and one water molecule. The Cu^II^ atom in the centrosymmetric [Cu(en)_2_(H_2_O)_2_]^2+^ cation lies on the inversion centre. The four N atoms of the ethylenediamine ligands in the equatorial plane around the Cu^II^ atom form a slightly distorted square-planar arrangement, while the slightly distorted Jahn-Teller octahedral coordination is completed by the two O atoms of water molecules in the axial positions (Table 1 and Fig. 1).

The Cu—N1 [2.015 (2) Å] and Cu—N2 [2.022 (2) Å] bond lengths and N1—Cu—N2 [84.91 (7)°] bond angle agree with those found in other [Cu(en)_2_(H_2_O)_2_]^2+^ complexes (Table 1). The Cu—O1W [2.503 (2) Å] bond is much longer than Cu—N bonds, as a result of the Jahn-Teller distortion. The Cu—OW bonds in other [Cu(en)_2_(H_2_O)_2_]^2+^ complexes are in the range of 2.416 (3)–2.693 (9) Å (Table 1). The value of the T parameter (Hathaway & Hodgson, 1973), which indicates the degree of tetragonal distortion about the Cu^II^ atom, is 0.81 and it agrees with the reported values, in the range of 0.76–0.84, for other [Cu(en)_2_(H_2_O)_2_]^2+^ complexes (Table 1).

In the crystal structure, the [Cu(en)_2_(H_2_O)_2_]^2+^ coordination cations and 3-(3-pyridyl)propionate anions are linked by O—H···O and N—H···O hydrogen-bonds (Table 2, Fig. 2) in parallel to the *a* axis. The amine H atom is linked to both carboxylate O atoms of 3-(3-pyridyl)propionate by three-centered/bifurcated N—H···O hydrogen-bonds (Jeffrey, 1997) and both of them form the *R*_1_^2^(4) ring motif (Bernstein *et al.*, 1995). The similar *R*_1_^2^(4) ring motifs with sulfonate or carboxylate groups are reported in[Cu(en)_2_(H_2_O)_2_](4-amino- naphthalene-1-sulfonate)_2_.2 H_2_O (Li *et al.*, 2005)and [Cu(en)_2_(H_2_O)_2_]- (*N*-carboxyglycinate).H_2_O (Kovbasyuk *et al.*, 1997).

The O—H···O hydrogen bonds between the coordinated water molecules and the carboxylate O atoms of 3-(3-pyridyl)propionate anions and N—H···O hydrogen bonds of amine H atoms form the *R*_2_^1^(6) and *R*_2_^2^(8) ring motifs (Bernstein *et al.*, 1995). These ring motifs are also reported for other [Cu(en)_2_(H_2_O)_2_]*X*_2_ complexes, while only the *R*_2_^2^(8) ring motifs are present in [Cu(en)_2_(H_2_O)_2_]*X*_2_ complexes, [where *X* = 4-chlorobenzoate (Lee *et al.*, 2005); *X* = 4-fluorobenzoate (Liu *et al.*, 2004), *X* = isonicotinate (Segla *et al.*, 2000) and *X* = 4-nitrobenzoate (Harrison *et al.*, 2007)]. On the other hand, the *R*_2_^1^(6) ring motifs are reported for [Cu(en)_2_(H_2_O)_2_]*X*_2_ complexes, [where *X* = naphthalene-2-sulfonate (Sharma *et al.*, 2005) and *X* = 2,6-dimethoxynicotinate (Jašková *et al.*, 2007)]. To the best of our knowledge, only [Cu(en)_2_(H_2_O)_2_](2-aminobenzoate)_2_ complex (Miminoshvili *et al.*, 2005), exhibits both *R*_2_^1^(6) and *R*_2_^2^(8) ring motifs, in the crystal structure.

The additional N—H···O hydrogen bonds form further *R*_2_^1^(6) ring motifs. Finally, two [Cu(en)_2_(H_2_O)_2_]^2+^ cations and two 3-(3-pyridyl)propionate anions are joined through *R*_4_^2^(8) ring motifs to form a layer parallel to the *a* axis. The layers of [Cu(en)_2_(H_2_O)_2_]^2+^ cations and 3-(3-pyridyl)- propionate anions are linked to form a three-dimensional network through uncoordinated water molecules by O—H···O and O—H···N hydrogen-bonds (Fig. 3).

The additional interactions between the 3-(3-pyridyl)propionate anions of (I) are the π-π stacking interactions (Janiak, 2000) between the two adjacent pyridine rings, (N3/C6—C10), with the centroid to centroid distances of C_g_···C_g_^v^ = 3.66 Å [symmetry code: (v) -*x*,-*y* + 2,-*z* + 2]. The distance between parallel planes of the stacked pyridine rings is 3.33 Å.

### Experimental

The violet [Cu(en)_2_(H_2_O)_2_](3-pypr)_2_.2H_2_O was formed in a methanolic solution of [Cu(3-pypr)_2_(H_2_O)_2_] (1.25 mmol) by adding ethylenediamine in the molar ratio of 1:2. The resulting solution was left to slowly evaporate at room temperature. In isolating the complex, it was necessary to add acetone to the concentrated solution. Well shaped violet crystals, suitable for X-ray structure analysis were collected after a few hours by filtration and finally dried *in vacuo* (yield; 90%). Anal. Calc. for C_20_H_40_N_6_O_8_; C, 43.20; H, 7.25; N, 15.11; Cu, 11.43. Found: C, 43.45; H, 7.47; N, 15.30; Cu, 11.21%. Selected IR data (cm^-1^): 1592 *versus*,br (ν_a_(COO^-^) + ν(C?N)); 1392 *versus* (ν_s_(COO^-^)); 605 s (δ(py), pyridine ring in-plane bending); 405 m (γ(py), pyridine ring out-of-plane bending). Electronic data (cm^-1^): 18 400 br.

### Refinement

H atoms were positioned geometrically, with O—H = 0.84 Å (for H_2_O), N—H = 0.92 Å (for NH_2_) and C—H = 0.95 and 0.99 Å for aromatic and methylene H, respectively, and constrained to ride on their parent atoms, with *U*_iso_(H) = *xU*_eq_(C,*N*,*O*), where *x* = 0.92 for O2W H and *x* = 1.2 for other H atoms.

### Crystal data

**Table:**

[Cu(C_2_H_8_N_2_)_2_(H_2_O_1_)_2_](C_8_H_8_NO_2_)_2_·2H_2_O	*Z* = 1
*M**_r_* = 556.13	*F*_000_ = 295
Triclinic, *P*1	*D*_x_ = 1.411 Mg m^−^^3^
Hall symbol: -P 1	Ag *K*α radiation λ = 0.56085 Å
*a* = 6.2620 (1) Å	Cell parameters from 2489 reflections
*b* = 8.5660 (2) Å	θ = 3.3–21.4º
*c* = 13.3550 (4) Å	µ = 0.47 mm^−^^1^
α = 75.271 (1)º	*T* = 153 (2) K
β = 83.809 (1)º	Prism, violet
γ = 70.863 (1)º	0.45 × 0.25 × 0.20 mm
*V* = 654.30 (3) Å^3^	

### Data collection

**Table:**

Bruker–Nonius KappaCCD diffractometer	2989 independent reflections
Radiation source: fine-focus sealed tube	2644 reflections with *I* > 2σ(*I*)
Monochromator: graphite	*R*_int_ = 0.079
*T* = 153(2) K	θ_max_ = 21.4º
ω and φ scans	θ_min_ = 3.3º
Absorption correction: numerical(HABITUS; Herrendorf & Bärnighausen, 1997)	*h* = −8→8
*T*_min_ = 0.808, *T*_max_ = 0.915	*k* = −11→11
15039 measured reflections	*l* = −17→17

### Refinement

**Table:**

Refinement on *F*^2^	Secondary atom site location: difference Fourier map
Least-squares matrix: full	Hydrogen site location: inferred from neighbouring sites
*R*[*F*^2^ > 2σ(*F*^2^)] = 0.041	H-atom parameters constrained
*wR*(*F*^2^) = 0.086	*w* = 1/[σ^2^(*F*_o_^2^) + (0.0215*P*)^2^ + 0.3896*P*] where *P* = (*F*_o_^2^ + 2*F*_c_^2^)/3
*S* = 1.07	(Δ/σ)_max_ < 0.001
2989 reflections	Δρ_max_ = 0.38 e Å^−^^3^
160 parameters	Δρ_min_ = −0.31 e Å^−^^3^
Primary atom site location: structure-invariant direct methods	Extinction correction: none

### Special details

**Table:**

Geometry. All e.s.d.'s (except the e.s.d. in the dihedral angle between two l.s. planes) are estimated using the full covariance matrix. The cell e.s.d.'s are taken into account individually in the estimation of e.s.d.'s in distances, angles and torsion angles; correlations between e.s.d.'s in cell parameters are only used when they are defined by crystal symmetry. An approximate (isotropic) treatment of cell e.s.d.'s is used for estimating e.s.d.'s involving l.s. planes.Least-squares planes (*x*,*y*,*z* in crystal coordinates) and deviations from them (* indicates atom used to define plane)2.3086 (0.0059) *x* + 8.2273 (0.0023) *y* - 0.2376 (0.0130) *z* = 6.3254 (0.0154)* 0.0032 (0.0016) N3* -0.0044 (0.0016) C6* 0.0010 (0.0017) C7* -0.0039 (0.0017) C8* 0.0003 (0.0018) C9* 0.0038 (0.0017) C103.3255 (0.0028) N3_$63.3331 (0.0028) C6_$63.3277 (0.0030) C7_$63.3326 (0.0031) C8_$63.3285 (0.0029) C9_$63.3249 (0.0030) C10_$6Rms deviation of fitted atoms = 0.0032
Refinement. Refinement of *F*^2^ against ALL reflections. The weighted *R*-factor *wR* and goodness of fit *S* are based on *F*^2^, conventional *R*-factors *R* are based on *F*, with *F* set to zero for negative *F*^2^. The threshold expression of *F*^2^ > σ(*F*^2^) is used only for calculating *R*-factors(gt) *etc*. and is not relevant to the choice of reflections for refinement. *R*-factors based on *F*^2^ are statistically about twice as large as those based on *F*, and *R*- factors based on ALL data will be even larger.

### Fractional atomic coordinates and isotropic or equivalent isotropic displacement parameters (Å^2^)

**Table:**

	*x*	*y*	*z*	*U*_iso_*/*U*_eq_	
Cu	0.5000	0.5000	0.5000	0.02500 (12)	
N1	0.5413 (3)	0.7274 (2)	0.48893 (14)	0.0328 (4)	
H1A	0.6147	0.7242	0.5459	0.039*	
H1B	0.6270	0.7540	0.4306	0.039*	
N2	0.1813 (3)	0.6372 (2)	0.45504 (14)	0.0307 (4)	
H2A	0.1410	0.5969	0.4047	0.037*	
H2B	0.0813	0.6297	0.5104	0.037*	
N3	−0.0294 (4)	0.8099 (3)	1.12361 (17)	0.0520 (6)	
O1	−0.0720 (3)	0.6040 (3)	0.66095 (13)	0.0493 (5)	
O2	−0.4121 (3)	0.7310 (2)	0.71927 (15)	0.0518 (5)	
O1W	0.3984 (3)	0.4843 (2)	0.68797 (15)	0.0515 (5)	
H1W	0.4422	0.5531	0.7088	0.063*	
H2W	0.2570	0.5084	0.6916	0.063*	
O2W	0.6770 (4)	0.9227 (3)	0.29173 (16)	0.0682 (6)	
H3W	0.7363	0.8948	0.2368	0.063*	
H4W	0.6086	1.0274	0.2809	0.063*	
C1	0.1767 (4)	0.8158 (3)	0.41381 (19)	0.0399 (5)	
H1C	0.0192	0.8928	0.4130	0.048*	
H1D	0.2402	0.8312	0.3421	0.048*	
C2	0.3163 (4)	0.8558 (3)	0.4831 (2)	0.0399 (5)	
H2C	0.3294	0.9709	0.4542	0.048*	
H2D	0.2442	0.8520	0.5530	0.048*	
C3	−0.2033 (4)	0.6638 (3)	0.72904 (17)	0.0339 (5)	
C4	−0.1063 (4)	0.6478 (3)	0.83208 (19)	0.0418 (5)	
H4A	−0.2050	0.6083	0.8890	0.050*	
H4B	0.0448	0.5608	0.8387	0.050*	
C5	−0.0846 (6)	0.8114 (3)	0.8436 (2)	0.0575 (8)	
H5A	−0.2360	0.8978	0.8392	0.069*	
H5B	0.0113	0.8527	0.7860	0.069*	
C6	0.0179 (4)	0.7906 (3)	0.94565 (19)	0.0389 (5)	
C7	−0.1118 (4)	0.8301 (3)	1.0313 (2)	0.0458 (6)	
H7	−0.2711	0.8749	1.0242	0.055*	
C8	0.1937 (5)	0.7467 (3)	1.1312 (2)	0.0507 (7)	
H8	0.2567	0.7298	1.1962	0.061*	
C9	0.3376 (4)	0.7045 (3)	1.0513 (2)	0.0460 (6)	
H9	0.4963	0.6603	1.0606	0.055*	
C10	0.2489 (4)	0.7271 (3)	0.95738 (19)	0.0417 (5)	
H10	0.3462	0.6992	0.9005	0.050*	

### Atomic displacement parameters (Å^2^)

**Table:**

	*U*^11^	*U*^22^	*U*^33^	*U*^12^	*U*^13^	*U*^23^
Cu	0.02142 (18)	0.02502 (19)	0.0296 (2)	−0.00597 (13)	−0.00336 (13)	−0.00873 (14)
N1	0.0370 (10)	0.0319 (10)	0.0332 (10)	−0.0132 (8)	−0.0058 (8)	−0.0086 (8)
N2	0.0266 (8)	0.0378 (10)	0.0292 (9)	−0.0082 (7)	−0.0023 (7)	−0.0121 (8)
N3	0.0777 (16)	0.0397 (12)	0.0397 (13)	−0.0190 (11)	0.0090 (11)	−0.0145 (10)
O1	0.0421 (9)	0.0819 (14)	0.0342 (9)	−0.0235 (9)	0.0035 (7)	−0.0280 (9)
O2	0.0401 (9)	0.0620 (12)	0.0555 (12)	−0.0036 (8)	−0.0106 (8)	−0.0300 (9)
O1W	0.0399 (9)	0.0668 (12)	0.0526 (11)	−0.0158 (8)	0.0015 (8)	−0.0243 (9)
O2W	0.0781 (14)	0.0525 (12)	0.0540 (13)	−0.0063 (10)	0.0207 (11)	−0.0053 (10)
C1	0.0364 (12)	0.0340 (12)	0.0404 (13)	0.0026 (9)	−0.0085 (10)	−0.0078 (10)
C2	0.0478 (13)	0.0284 (11)	0.0425 (14)	−0.0068 (10)	−0.0010 (10)	−0.0129 (10)
C3	0.0380 (12)	0.0386 (12)	0.0311 (12)	−0.0163 (9)	−0.0034 (9)	−0.0119 (9)
C4	0.0532 (14)	0.0418 (13)	0.0336 (13)	−0.0141 (11)	−0.0079 (10)	−0.0126 (10)
C5	0.089 (2)	0.0386 (14)	0.0508 (17)	−0.0225 (14)	−0.0349 (15)	−0.0035 (12)
C6	0.0540 (14)	0.0294 (11)	0.0378 (13)	−0.0159 (10)	−0.0144 (11)	−0.0068 (10)
C7	0.0436 (13)	0.0338 (12)	0.0636 (18)	−0.0142 (10)	−0.0036 (12)	−0.0137 (12)
C8	0.083 (2)	0.0384 (14)	0.0338 (14)	−0.0169 (13)	−0.0209 (13)	−0.0078 (11)
C9	0.0479 (14)	0.0426 (14)	0.0516 (16)	−0.0146 (11)	−0.0149 (12)	−0.0121 (12)
C10	0.0519 (14)	0.0419 (13)	0.0353 (13)	−0.0169 (11)	−0.0005 (10)	−0.0132 (10)

### Geometric parameters (Å, °)

**Table:**

Cu—O1W^i^	2.503 (2)	C1—C2	1.506 (3)
Cu—O1W	2.503 (2)	C1—H1C	0.9900
Cu—N1^i^	2.015 (2)	C1—H1D	0.9900
Cu—N1	2.015 (2)	C2—H2C	0.9900
Cu—N2^i^	2.022 (2)	C2—H2D	0.9900
Cu—N2	2.022 (2)	C3—C4	1.521 (3)
O1—C3	1.251 (3)	C4—C5	1.498 (3)
O2—C3	1.250 (3)	C4—H4A	0.9900
O1W—H1W	0.84	C4—H4B	0.9900
O1W—H2W	0.84	C5—C6	1.513 (3)
O2W—H3W	0.84	C5—H5A	0.9900
O2W—H4W	0.84	C5—H5B	0.9900
N1—C2	1.472 (3)	C6—C7	1.378 (4)
N1—H1A	0.9200	C6—C10	1.379 (3)
N1—H1B	0.9200	C7—H7	0.9500
N2—C1	1.480 (3)	C8—C9	1.363 (4)
N2—H2A	0.9200	C8—H8	0.9500
N2—H2B	0.9200	C9—C10	1.370 (3)
N3—C8	1.327 (4)	C9—H9	0.9500
N3—C7	1.337 (4)	C10—H10	0.9500
			
O1W^i^—Cu—O1W	180.0	H1C—C1—H1D	108.5
N1^i^—Cu—O1W^i^	88.72 (7)	N1—C2—C1	107.48 (18)
N1—Cu—O1W^i^	91.28 (7)	N1—C2—H2C	110.2
N2^i^—Cu—O1W^i^	93.19 (6)	C1—C2—H2C	110.2
N2—Cu—O1W^i^	86.81 (6)	N1—C2—H2D	110.2
N1^i^—Cu—O1W	91.28 (7)	C1—C2—H2D	110.2
N1—Cu—O1W	88.72 (7)	H2C—C2—H2D	108.5
N2^i^—Cu—O1W	86.81 (6)	O2—C3—O1	124.1 (2)
N2—Cu—O1W	93.19 (6)	O2—C3—C4	117.4 (2)
N1^i^—Cu—N1	180.00 (11)	O1—C3—C4	118.4 (2)
N1^i^—Cu—N2^i^	84.91 (7)	C5—C4—C3	113.0 (2)
N1—Cu—N2^i^	95.09 (7)	C5—C4—H4A	109.0
N1^i^—Cu—N2	95.09 (7)	C3—C4—H4A	109.0
N1—Cu—N2	84.91 (7)	C5—C4—H4B	109.0
N2^i^—Cu—N2	180.0	C3—C4—H4B	109.0
C2—N1—Cu	108.14 (13)	H4A—C4—H4B	107.8
C2—N1—H1A	110.1	C4—C5—C6	111.8 (2)
Cu—N1—H1A	110.1	C4—C5—H5A	109.2
C2—N1—H1B	110.1	C6—C5—H5A	109.2
Cu—N1—H1B	110.1	C4—C5—H5B	109.2
H1A—N1—H1B	108.4	C6—C5—H5B	109.2
C1—N2—Cu	107.44 (13)	H5A—C5—H5B	107.9
C1—N2—H2A	110.2	C7—C6—C10	116.7 (2)
Cu—N2—H2A	110.2	C7—C6—C5	122.5 (2)
C1—N2—H2B	110.2	C10—C6—C5	120.8 (2)
Cu—N2—H2B	110.2	N3—C7—C6	124.6 (2)
H2A—N2—H2B	108.5	N3—C7—H7	117.7
C8—N3—C7	116.3 (2)	C6—C7—H7	117.7
Cu—O1W—H1W	110.3	N3—C8—C9	123.8 (2)
Cu—O1W—H2W	104.9	N3—C8—H8	118.1
H1W—O1W—H2W	111.9	C9—C8—H8	118.1
H3W—O2W—H4W	111.2	C8—C9—C10	118.7 (2)
N2—C1—C2	107.81 (18)	C8—C9—H9	120.7
N2—C1—H1C	110.1	C10—C9—H9	120.7
C2—C1—H1C	110.1	C9—C10—C6	119.8 (2)
N2—C1—H1D	110.1	C9—C10—H10	120.1
C2—C1—H1D	110.1	C6—C10—H10	120.1
			
N2^i^—Cu—N1—C2	−165.34 (14)	O1—C3—C4—C5	104.8 (3)
N2—Cu—N1—C2	14.66 (14)	C3—C4—C5—C6	−178.5 (2)
O1W^i^—Cu—N1—C2	101.34 (14)	C4—C5—C6—C7	−96.9 (3)
O1W—Cu—N1—C2	−78.66 (14)	C4—C5—C6—C10	81.8 (3)
N1^i^—Cu—N2—C1	−165.31 (14)	C8—N3—C7—C6	−0.2 (4)
N1—Cu—N2—C1	14.70 (14)	C10—C6—C7—N3	−0.5 (4)
O1W^i^—Cu—N2—C1	−76.88 (14)	C5—C6—C7—N3	178.2 (2)
O1W—Cu—N2—C1	103.12 (14)	C7—N3—C8—C9	0.7 (4)
Cu—N2—C1—C2	−40.8 (2)	N3—C8—C9—C10	−0.4 (4)
Cu—N1—C2—C1	−40.7 (2)	C8—C9—C10—C6	−0.4 (4)
N2—C1—C2—N1	54.5 (2)	C7—C6—C10—C9	0.8 (3)
O2—C3—C4—C5	−77.7 (3)	C5—C6—C10—C9	−178.0 (2)

Symmetry codes: (i) −*x*+1, −*y*+1, −*z*+1.

### Hydrogen-bond geometry (Å, °)

**Table:**

*D*—H···*A*	*D*—H	H···*A*	*D*···*A*	*D*—H···*A*
N1—H1A···O2^ii^	0.92	2.32	3.130 (3)	147
N1—H1A···O1^ii^	0.92	2.41	3.247 (2)	152
N1—H1B···O2W	0.92	2.11	2.944 (3)	151
N2—H2A···O1^iii^	0.92	2.29	3.150 (3)	155
N2—H2B···O1	0.92	2.12	3.019 (2)	164
O1W—H1W···O2^ii^	0.84	2.06	2.873 (3)	164
O1W—H2W···O1	0.84	2.00	2.814 (2)	164
O2W—H3W···N3^iv^	0.84	2.08	2.899 (3)	163
O2W—H4W···O2^v^	0.84	2.03	2.859 (3)	169

Symmetry codes: (ii) *x*+1, *y*, *z*; (iii) −*x*, −*y*+1, −*z*+1; (iv) *x*+1, *y*, *z*−1; (v) −*x*, −*y*+2, −*z*+1.

### Table 2 Comparative geometrical parameters (Å, °) and T parameter for selected trans-[Cu(en)_2_(H_2_O)_2_] X_2_ complexes

**Table:**

X_2_	Cu–N1	Cu–N2	Cu–OW	*T*^a^	N1–Cu–N2
(3-pypr)_2_.2H_2_O	2.015 (2)	2.022 (2)	2.503 (2)	0.81	84.91 (7)
(2,6-(MeO)_2_nic)_2_^b^	2.018 (2)	2.020 (2)	2.467 (2)	0.82	83.96 (8)
(4-O_2_Nbz)_2_^c^	2.015 (2)	2.027 (2)	2.537 (2)	0.80	84.92 (7)
(2-NH_2_bz)_2_^d^	2.012 (4)	2.020 (5)	2.503 (4)	0.81	84.52 (9)
(benz)_2_^e^	2.009 (4)	2.017 (4)	2.653 (5)	0.76	84.8 (2)
(isonic)_2_^f^	2.021 (2)	2.018 (2)	2.598 (2)	0.78	84.98 (6)
(4-Fbz)_2_^g^	2.018 (4)	2.021 (4)	2.579 (4)	0.78	85.0 (2)
(naphs)_2_^h^	2.020 (2)	2.018 (2)	2.513 (1)	0.80	84.63 (7)
(stz)_2_.2H_2_O^i^	2.042 (3)	2.033 (3)	2.484 (3)	0.82	84.1 (1)
F_2_.4H_2_O^j^	2.019 (5)	2.023 (5)	2.571 (6)	0.78	84.6 (2)
(NH_2_naphs)_2_.2H_2_O^k^	2.023 (2)	2.012 (2)	2.437 (2)	0.83	84.30 (9)
(Clbtts)_2_^l^	2.019 (4)	2.049 (3)	2.416 (3)	0.84	84.2 (2)
(Cl-Fbz)_2_^m^	2.009 (2)	2.016 (2)	2.618 (1)	0.77	84.30 (6)
(BPh_4_)_2_.2DMSO^n^	2.022 (10)	2.055 (8)	2.693 (9)	0.76	84.9 (4)
(edcarb).2H_2_O^o^	1.996 (2)	2.022 (2)	2.556 (2)	0.79	84.78 (7)

(a) The value of the *T* parameter (*T* = *R*_S_/*R*_L_), indicating the degree of tetragonal distortion about the Cu^II^ atom (Hathaway & Hodgson, 1973); (b) Jašková *et al.* (2007) [2,6-(MeO)_2_nic is 2,6-dimethoxynicotinate]; (c) Harrison *et al.* (2007) [4-O_2_Nbz is 4-nitrobenzoate]; (d) Miminoshvili *et al.* (2005) [2-NH_2_bz^c^ is 2-aminobenzoate]; (e) Carballo *et al.* (2005) [benz is benzilate]; (f) Segla *et al.* (2000) [isonic is isonicotinate]; (g) Liu *et al.* (2004) [4-Fbz is 4-fluorobenzoate]; (h) Sharma *et al.* (2005) [naphs is naphthalene-2-sulfonate]; (i) Anacona *et al.* (2002) [stz is sulfathiazole]; (j) Emsley *et al.* (1988; 1990); (k) Li *et al.* (2005) [NH_2_naphs is 4-aminonaphthalene-1-sulfonate]; (l) Gonzalez-Alvarez *et al.* (2003) [Clbtts is N-2-(6-chlorobenzothiazole)toluenesulfonamide]; (m) Lee *et al.* (2005); (n) Mahadevan *et al.* (1986); (o) Kovbasyuk *et al.* (1997) [edcarb is ethylene-1,2-dicarbonate].
